# State-of-the-Art Medical Imaging Supporting Supermicrosurgery: A Scoping Review, Practical Guidelines, and Illustrative Case Report

**DOI:** 10.1055/a-2798-0198

**Published:** 2026-03-27

**Authors:** Jos Velleman, Jin Geun Kwon, Changsik John Pak, Hyunsuk Peter Suh, Joon Pio Hong

**Affiliations:** 1 Department of Plastic, Reconstructive and Aesthetic Surgery, University Hospitals Leuven, Belgium; 2 Department of Plastic and Reconstructive Surgery, Asan Medical Center, Seoul, South Korea

**Keywords:** medical imaging, supermicrosurgery, reconstructive surgery, extremity/lymphedema, microsurgery, research/experimental, imaging

## Abstract

In the era of supermicrosurgery, clinicians need more precise imaging modalities to know the exact microvascular and lymphatic anatomy of the patient. The goal of this review is to answer the research question “What are the state-of-the-art medical imaging modalities supporting supermicrosurgery?”

This study is a scoping review and case report.

Computed tomography angiography (CTA) and magnetic resonance angiography (MRA) are well-known current standard imaging modalities in flap surgery. In lymphatic surgery, lymphoscintigraphy is the gold standard. (Ultra)high-frequency ultrasound (UHFUS) has taken a major role in preoperative planning of flap surgery and lymphatic surgery. Practical guidelines on the use of UHFUS in flap planning and lymphatic surgery are described in this review article. Indocyanine green angiography and near-infrared fluorescent lymphography have also become key elements in modern flap surgery and lymphatic surgery. Moreover, contrast-enhanced magnetic resonance lymphography produces high-resolution imaging of superficial as well as deep lymphatic vessels. Laser tomography and photoacoustic imaging are promising experimental imaging techniques in lymphatic surgery.

This review article describes and compares possible imaging modalities for preoperative planning and intraoperative guidance with the aims of enhancing surgical outcomes, reducing operative time, and preventing complications in supermicrosurgery. Moreover, a case report is described in order to illustrate the practical imaging work-up in daily practice.

## Introduction

Imaging technology has become indispensable in modern plastic surgery, particularly in the fields of flap reconstructions and microsurgery. These advanced imaging modalities provide invaluable assistance in preoperative planning, intraoperative navigation, and postoperative monitoring, aiming to improve surgical outcomes, minimize operation time, and avoid complications. However, in the rising era of supermicrosurgery, with perforator to perforator anastomoses and lymphaticovenular anastomoses, clinicians need even more precise imaging modalities in order to know the exact microvascular and lymphatic anatomy of the patient. 1 Therefore, this review article gives a detailed description of the latest imaging modalities specifically supporting supermicrosurgery. Moreover, the article provides practical guidelines and compares the different imaging modalities for preoperative planning and intraoperative guidance in the field of supermicrosurgery. Furthermore, a case report is described in order to illustrate the imaging work-up for supermicrosurgery in daily practice.

## Methods

The research question “What are the state-of-the-art medical imaging modalities supporting supermicrosurgery?” was answered by performing a scoping review and by describing a case report. The search terms “medical imaging,” “supermicrosurgery,” “perforator flap,” and “lymphedema” were used in the databases PubMed and Cochrane Library. The keyword “medical imaging” was used with the Boolean operator “and” in combination with the other search terms: “medical imaging” and “supermicrosurgery,” “medical imaging” and “perforator flap,” “medical imaging” and “lymphedema,” searching for publications in English. The literature search was executed from October 2024 to March 25, 2025. A total of 32 articles were selected. Informed consent was obtained from the patient for the use of the medical record and pictures in the case report.

## Results

### Imaging Modalities

Computed tomography angiography (CTA) and magnetic resonance angiography (MRA) are considered to be current standard imaging modalities in flap surgery. 2 Ultrasound, indocyanine green (ICG), and smartphone-based thermal imaging are novel players in the field of imaging for flap surgery. 2 In lymphatic surgery, lymphoscintigraphy is the gold standard. 2 Contrast-enhanced magnetic resonance lymphography (MRL), (ultra)high-frequency ultrasound (UHFUS), near-infrared fluorescent lymphography, photoacoustic imaging, and laser tomography are more recent imaging techniques for lymphatic surgery. 2

#### Computed Tomography Angiography and Magnetic Resonance Angiography

CTA is considered the gold standard for preoperative imaging in flap surgery, visualizing the flap anatomy as well as the recipient vessels. 2 Use of CTA can decrease the total operative time and improve outcomes in microsurgical procedures. 3 CTA is accessible in numerous medical centers. It provides imaging of perforators and enables visualization of vessels as small as 0.3 mm. 4 Moreover, three-dimensional (3D) reconstructions can be created. Furthermore, CTA is able to identify additional conditions, such as incidentalomas, metastatic diseases, and abdominal wall abnormalities. 5 However, the use of intravenous iodinated contrast has an associated risk of nephrotoxicity. 6

**Fig. 1:**
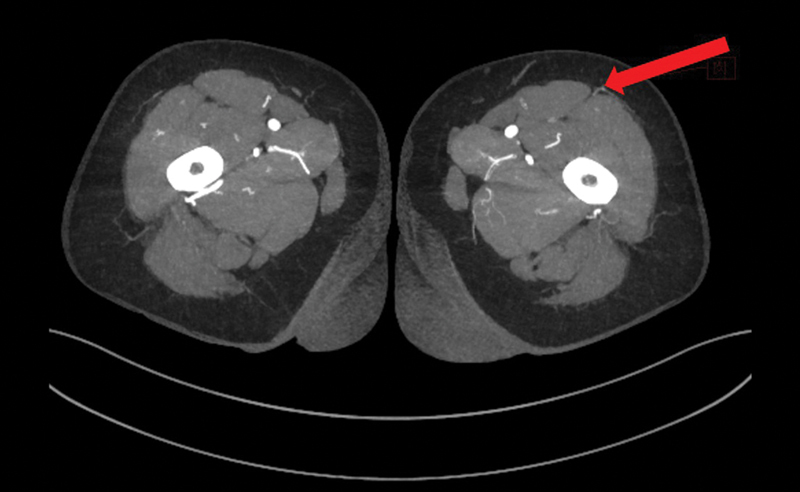
Computed tomography angiography. The red arrow shows a perforator of the descending branch of the lateral femoral circumflex artery.

**Fig. 2:**
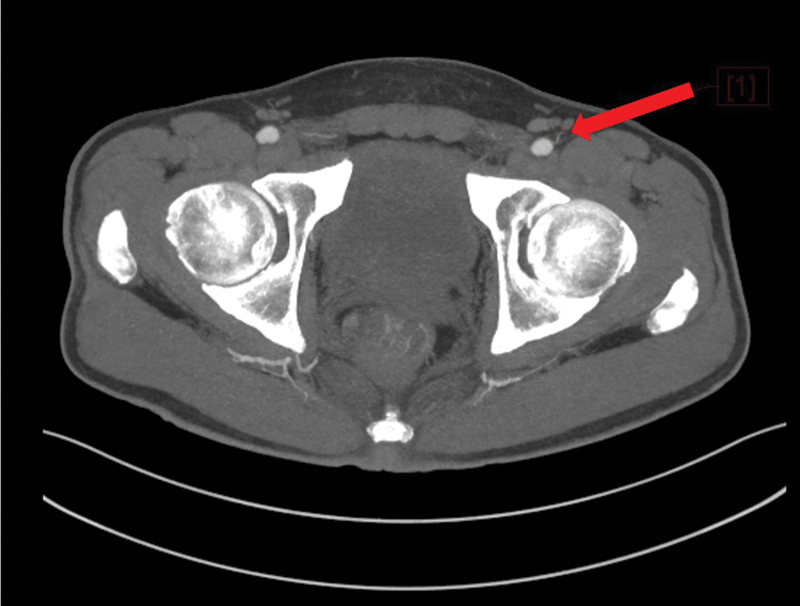
Computed tomography angiography. The red arrow shows the superficial circumflex iliac artery, with a deep and a superficial branch.

MRA offers an alternative to CTA. MRA avoids radiation exposure. Additionally, gadolinium-based contrast agents have a lower likelihood of triggering an acute allergic reaction (0.07%) compared to radioactive agents (3%). 7 8 However, the imaging process of MRA is more time-consuming, more expensive, and has a higher risk of motion artefacts than CTA. 9 MRA can visualize vessels with a minimum diameter of approximately 0.8 mm, compared to the 0.3 mm in CTA 10 11 12 ( Figs. 1 and 2 ).

#### Color Doppler Ultrasonography

In the field of supermicrosurgery, including flap surgery and lymphatic surgery, color Doppler ultrasonography (CDU) has taken a major role in preoperative planning. 13 It offers several advantages compared with handheld Doppler devices, which are limited by lower accuracy and an inability to visualize vessels or lymphatics. 9 CDU offers real-time, in-depth visual information about tissue characteristics, perforator size, vessel course, and flow velocity. 14 The color mode displays the direction of the blood flow. The frequency of transducers for perforator mapping typically ranges from 5 to 12 MHz in conventional ultrasound devices, between 15 and 20 MHz in high-frequency probes, and up to 70 MHz in ultrahigh-frequency ultrasound (UHFUS) probes. 14 15 16 Probes with a higher frequency show a better resolution, which allows for the visualization of small vascular structures. However, the frequency of the probe is inversely correlated to the depth of the visualized tissue. 14 By using this wide range of frequencies as well as the color mode, the clinician can map the whole vascular anatomy of the flap and recipient site: The major vessels, perforators, up to the subdermal capillaries. 14 This creates a 3D view of the vessels and surrounding tissues and enables assessment of the flow in the vessels. 14 These features allow a detailed preoperative mapping of the vasculature of superthin skin flaps as well as a clear view of the perforators in the recipient site in case of perforator-to-perforator anastomoses. 17 18 Moreover, high-frequency and UHFUS can detect lymphatic vessels and surrounding venules based on shape, echogenic texture, color mode, collapsibility, and location. 19 20

##### Practical Guidelines for the Use of Color Doppler Ultrasonography in Flap Planning

The practical use of CDU in flap planning can be illustrated by the planning of an anterolateral thigh (ALT) perforator flap. Kehrer et al described this process step by step. 21 Perforator mapping is performed the day before the free flap surgery and is affirmed in the start of the surgery. Mapping should be done in the same position as the surgery. In case of the ALT flap, this is supine with the foot perpendicular to the table. Classical marking of the intermuscular septum between the rectus femoris muscle and the vastus lateralis muscle is performed by drawing the line between the anterior superior iliac spine and the mid-upper boundary of the patella. Then, the 3-cm radius circle at the midpoint of this line is marked. A marking is placed at the point 5 cm distal from a mid-inguinal point on the line representing the intermuscular septum. Around this point, the lateral circumflex femoral artery branched off the profunda femoris artery. The distance from this last marking to ALT perforators estimates the possible pedicle length.

An ultrasound device with multifrequency linear transducers of 5 to 20 MHz is frequently used. 22 The search for appropriate ALT perforators starts in brightness mode (B-mode). The probe is placed perpendicular to the thigh axis in the middle of the target circle. Skin, subcutaneous fat, superficial fascia, deep fascia, muscle, intermuscular septum, and femur are traced. The skin is the most superficial hyperechoic (very bright) thin layer. Down to the skin, the subcutaneous fat can be identified as a dark layer, isoechogenic, or slightly hyperechoic compared to the skeletal muscle. The superficial fascia is a bright band separating the dark superficial and the dark deep fat layer. Next, the deep fascia is found as a thick hyperechoic (bright) layer between the subcutaneous fat and the muscle. The skeletal muscle shows dark on ultrasound and contains multiple tiny layers. Active contraction of the muscle can be visualized. Between the rectus femoris muscle and the vastus lateralis muscle, the bright intermuscular septum is found. It runs from medial-deep to lateral-superficial (oblique). Deeper, the vastus intermedius muscle and subsequently, the femur are identified. The femur appears bright with a posterior acoustic cancellation (black). The gain (the amplification in signal strength produced by the amplifier), focus, and depth are adjusted in order to position the deep fascia in central in the image.

**Fig. 3:**
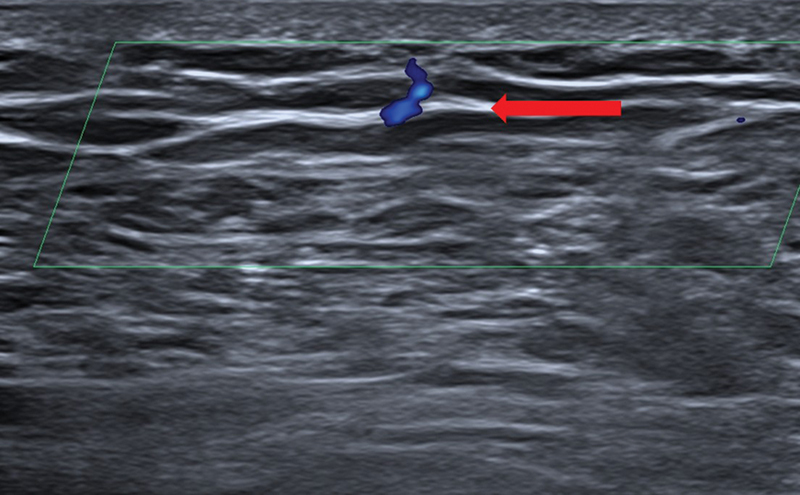
Color Doppler ultrasonography. A linear 24 MHz probe was used. The red arrow shows the superficial fascia. Here, the perforator is piercing the superficial fascia.

Next, the perforators and source vessels can be searched. Marking the points where the perforators ≥0.5 mm in diameter pierce the deep and superficial fascia is an important goal of the mapping. The emergence point of a perforator through the deep fascia can be seen as an interruption in the deep fascia. Color flow mode helps with further visualization of the blood vessels. The color box is usually tilted by the use of the steer button, which alters the angle of the ultrasound beam with respect to the transducer. Thereby, the angle between the ultrasound beam and the axis of flow in the blood vessel should be between 45 and 60 degrees. 23 If the flow in the blood vessel runs toward the transducer, the color is coded red, and if the flow in the blood vessel runs away from the transducer, the color is coded blue. The probe is moved distally and proximally within the target circle. Attention is paid to the medial border of the vastus lateralis muscle. The presence of color in the area of the deep fascia may indicate a perforator. This should be correlated with the mergence point of a perforator through the deep fascia found in B-mode. Once a perforator is located, tilting the probe and sliding in multiple directions allow to follow the perforator's course from the skin through subcutis, piercing the superficial fascia, subsequently piercing the deep fascia, and showing a septal or intramuscular course up to the source vessel. In the case of supermicrosurgery, aiming for perforator-to-perforator anastomosis, the mapping only locates the perforator. When a longer pedicle is needed, the source vessel is located as well. The mergence points of a perforator through the deep and superficial fascia are marked on the skin (marking in the middle of the probe once the mergence point is visualized in the middle of the screen). Depending on the plane of elevation, one of these points will be the area where the perforator is encountered when dissecting the flap. In case of a pure skin perforator flap, probes with frequencies over 20 MHz are useful visualize the vasculature of the skin. Turning the probe 90 degrees gives an additional longitudinal view. Further details can be found in the article by Kehrer et al 21 ( Figs. 3 4 5 6 ).

**Fig. 4:**
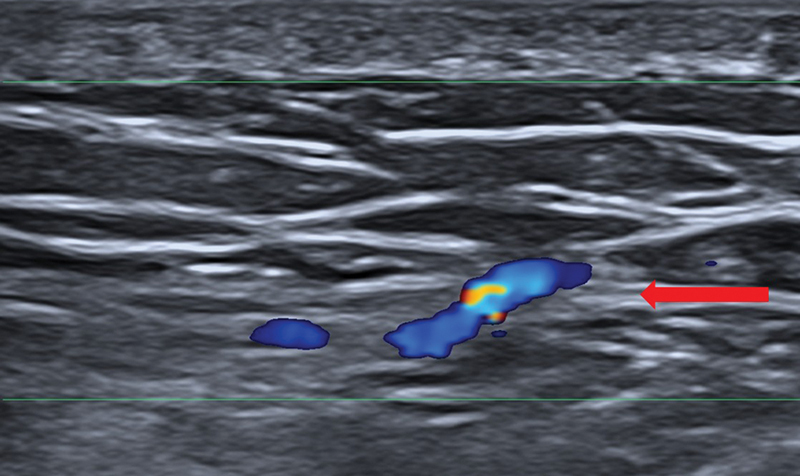
Color Doppler ultrasonography. A linear 24 MHz probe was used. The red arrow shows the deep fascia. Here, the perforator is piercing the deep fascia.

**Fig. 5:**
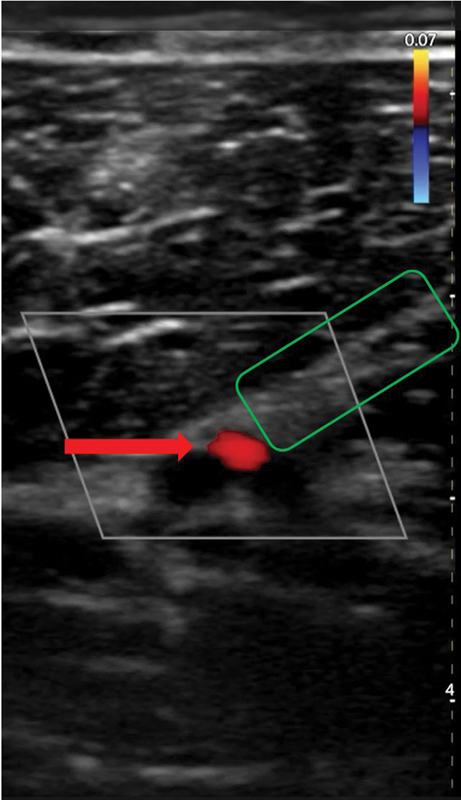
Color Doppler ultrasonography. A wireless handheld 12 MHz device was used. The red arrow shows the descending branch of the lateral femoral circumflex artery. The green box shows the septum dividing the vastus lateralis and the rectus femorismuscles.

**Fig. 6:**
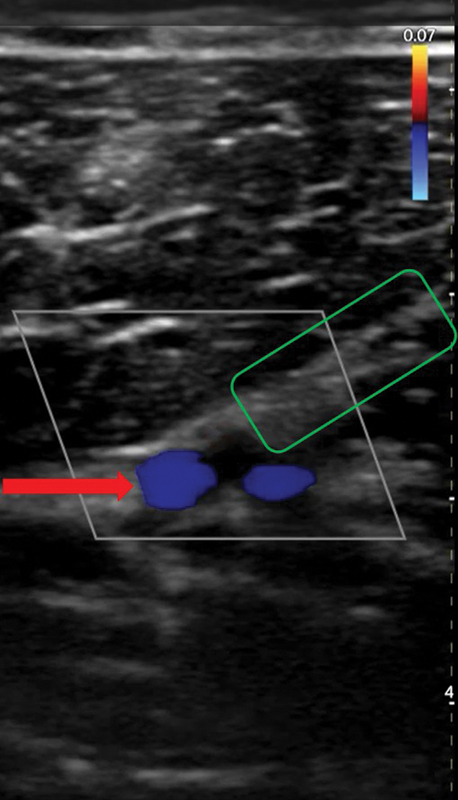
Color Doppler ultrasonography. A wireless handheld 12 MHz device was used. The red arrow shows the two concomitant veins. The green box shows the septum dividing the vastus lateralis and the rectus femoris muscles.

##### Practical Guidelines for the Use of Color Doppler Ultrasonography in Recipient Vessel Selection

The article by Hong et al gives a detailed description of the use of CDU in the selection of recipient vessels. 22 Linear transducers of 5 to 20 MHz are often used to identify the recipient vessels. Information concerning the anatomy of the recipient vessels can be retrieved: Location, diameter, pathway, an overall overview of the branching pattern, as well as detailed information on perforators branching from the source vessel. Perforators with good pulsations near the defect can be used as recipient vessels. Moreover, superficial veins around the defect can also be used as recipient veins. Furthermore, information on the physiological aspect, like the velocity of the recipient artery, can be retrieved. Main arteries of the lower leg (peroneal, posterior, and anterior tibial artery) show a normal range of peak arterial velocity of 55 ± 10 cm/s. The arteries of the forearm have a normal range of peak arterial velocity of 50 ± 15 cm/s. The average peak systolic velocity of the superior thyroid artery is 17 ± 5 cm/s. The use of a perforator as a recipient vessel in ischemic diabetic foot patients was studied by Suh et al. 24 25 It was found that the recipient artery is safe to use when the velocity is over 15 to 20 cm/s. 24 25 If the velocity is lower than the normal range, the cause should be searched for and resolved if possible.

CDU can provide this in-depth information on the anatomy and physiology of recipient vessels. However, it is difficult to produce an overall view of, for example, the whole limb. Especially in cases of trauma, chronic wounds, and oncologic reconstructions, CTA or a conventional angiogram is advised if the flow of the major axial artery is unclear.

Furthermore, it is important to take into account the concept of the zone of injury, known as a thrombogenic zone that extends beyond the macroscopically evident damaged zone. CDU can evaluate possible recipient vessels within the zone of injury by measuring the flow velocity and by examining if the surrounding tissue is supple or scarred. However, the most important factors in selecting the recipient artery may be the direct visualization of strong pulsations and adequate outflow after opening the vessel during the operative exploration. Therefore, it can still be necessary to search for recipient vessels proximal or distal to the zone during surgery, despite the preoperative CDU examination.

##### Practical Guidelines for the Use of Ultrasound in Planning of Lymphatic Surgery

Lymphatic vessels can be visualized by high-frequency ultrasound probes with an upper frequency of 15 to 18 MHz. However, UHFUS provides more accurate imaging of the lymphatic vessels. 15 The use of ultrasound addresses limitations of ICG lymphography by enabling the detection of lymphatic vessels that are hidden by dermal backflow or lymphatic vessels situated in deeper tissue layers. 15 UHFUS is able to measure the size of lymphatic vessels. 15 19 Moreover, UHFUS makes it possible to evaluate the thickness of the lymphatic vessel wall in relation to the lumen in order to estimate the degree of lymphosclerosis. 14

**Fig. 7:**
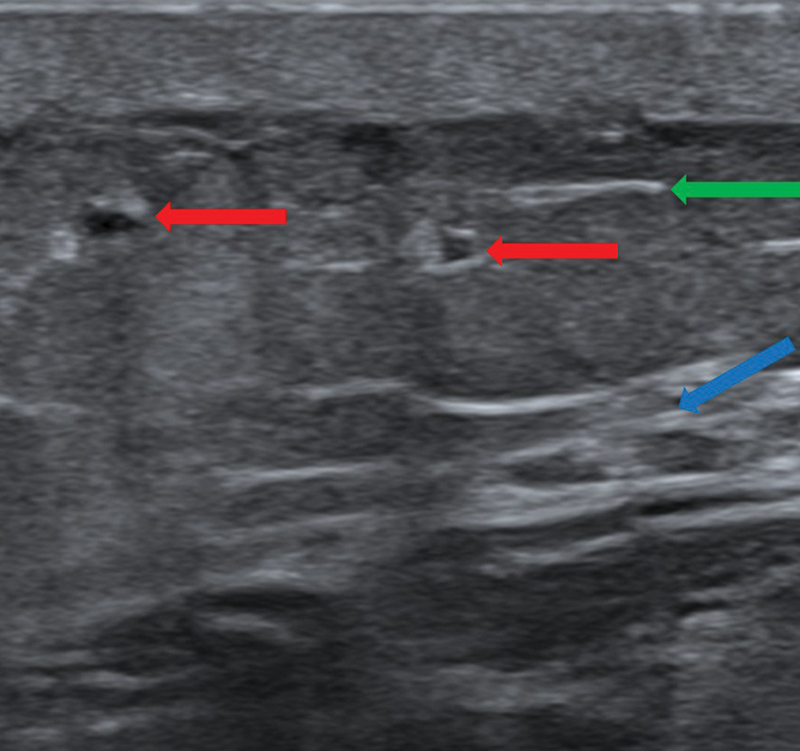
Ultrasonography of lymphatic vessels. A 22 MHz hockey stick probe was used. Red arrows show the lymphatic vessels. The green arrow shows the superficial fascia. The blue arrow shows the deep fascia.

The majority of lymphatic vessels are found below the superficial fascia. 26 The other characteristics of lymphatic vessels in ultrasonography are described as intermittent homogeneous, hypoechoic, and specular misshapen images in sagittal B-mode; no colors visible with color flow mode, and no convergence with the artery, the vein, or the nerve. 26 27 When the transducer is pushed against the skin, the lymphatic vessels have little tendency to collapse in comparison with the easily collapsing veins 15 ( Figs. 7 8 9 ).

**Fig. 8:**
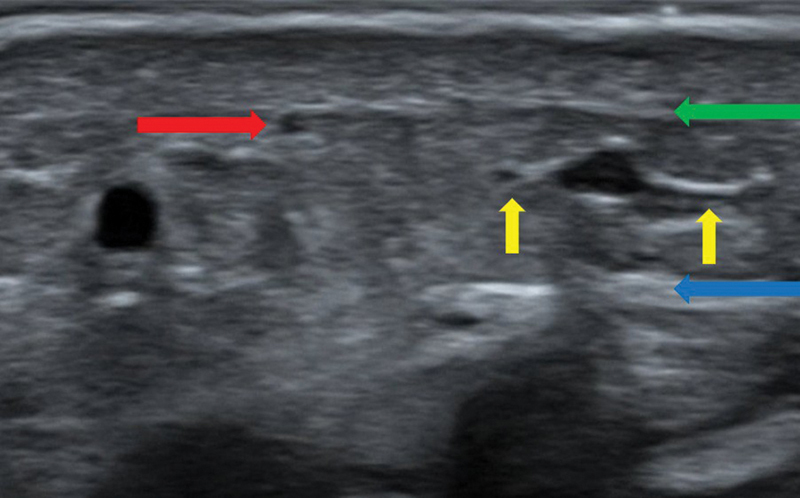
Ultrasonography of lymphatic vessels and veins. A 22 MHz hockey stick probe was used. The red arrow shows the lymphatic vessel. The green arrow shows the superficial fascia. The blue arrow shows the deep fascia. The yellow arrows show two smaller side branches of the vein. Those are a good choice for performing a lymphovenous anastomosis, using the Venturi effect. 28

**Fig. 9:**
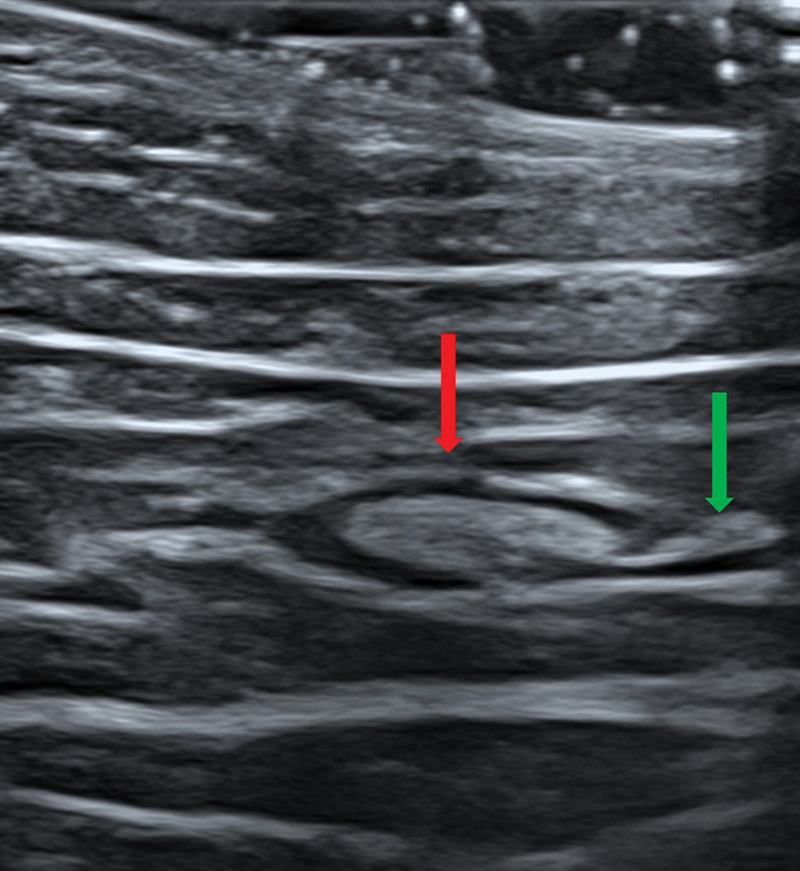
Ultrasonography of a lymph node (groin). A 22 MHz hockey stick probe was used. The red arrow shows the lymph node. The green arrow shows the efferent lymphatic vessel.

#### Indocyanine Green Angiography and Near-Infrared Fluorescent Lymphography

##### Indocyanine Green Angiography

ICG is administered intravenously in order to visualize the perfusion of tissues. 2 It binds to albumin. Application of near-infrared light makes the ICG fluorescent. 2 By using the imaging equipment, the fluorescence is detected through 5- to 10-mm-thick tissue. 2 If administered intravenously, perfusion of the flap, patency of the anastomosis, as well as perfusion of mastectomy skin or soft tissues after trauma can be evaluated peroperatively.

##### Near-Infrared Fluorescent Lymphography

**Fig. 10:**
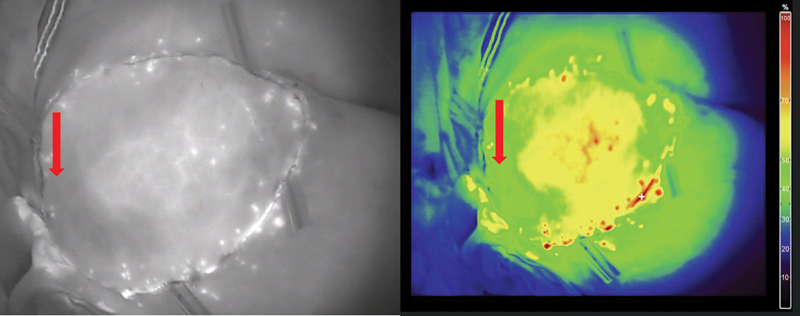
After intravenous injection of ICG, perfusion of the flap reconstruction is evaluated. The red arrows show a zone of reduced perfusion caused by local pressure of the underlying humeral head on the flap. ICG, indocyanine green.

ICG is administered intradermally in order to visualize lymphatic vessels in real time. 2 Lymphatic patterns can be recognized: Linear in functional lymphatics and splash, stardust, and diffuse patterns in degenerated lymphatics. 2 29 Moreover, after performing a lymphovenous anastomosis, patency can be tested by ICG. The near-infrared fluorescent lymphography is also a tool to visualize the lymphatic vessels during the planning of a lymphatic flow-through (LyFT) flap 30 ( Figs. 10 11 12 13 ).

**Fig. 11:**
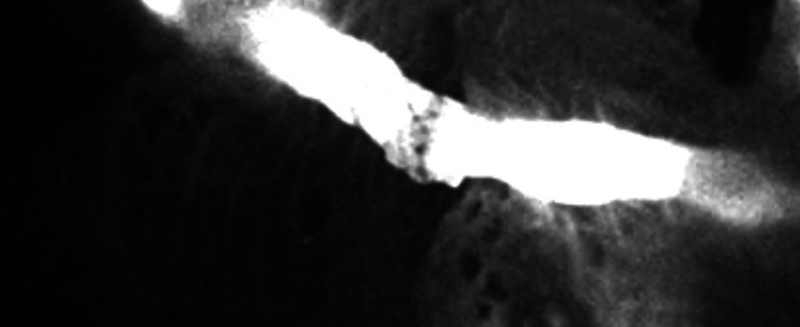
After intravenous injection of ICG, the patency of the microanastomosis is confirmed. ICG, indocyanine green.

**Fig. 12:**
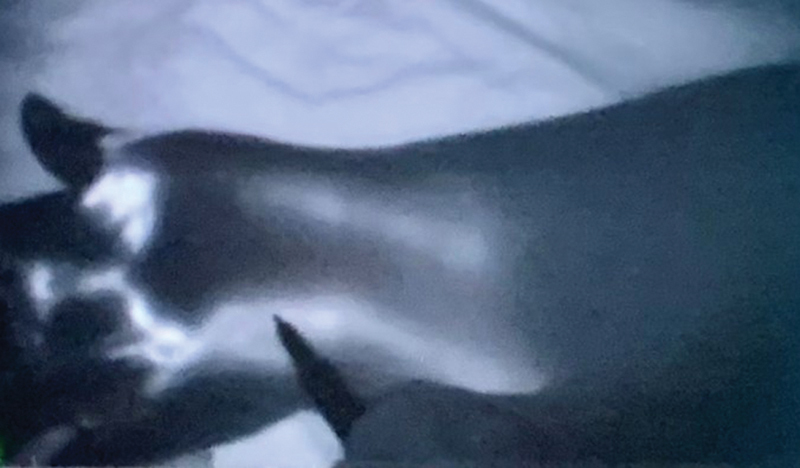
After intradermal injection of ICG (web spaces), the lymphatic vessels are visualized and marked on the dorsal forearm. ICG, indocyanine green.

**Fig. 13:**
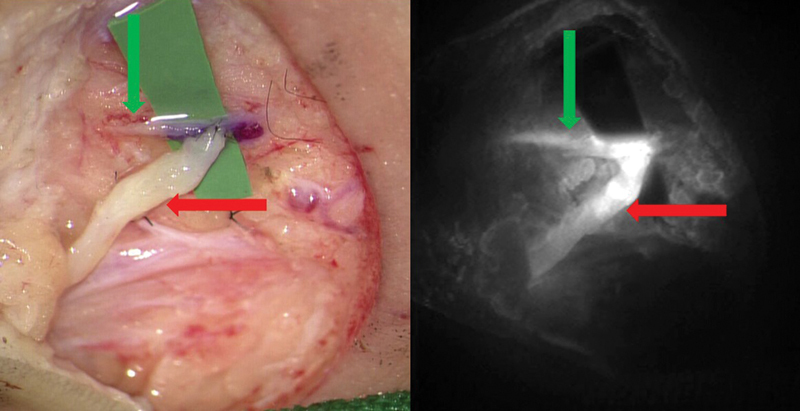
After intradermal injection of ICG, the patency of the side-to-end lymphovenous supermicroanastomosis is confirmed. The green arrows show the lymphatic vessel and the red arrows show the vein. ICG, indocyanine green.

#### Smartphone-Based Thermal Imaging

**Fig. 14:**
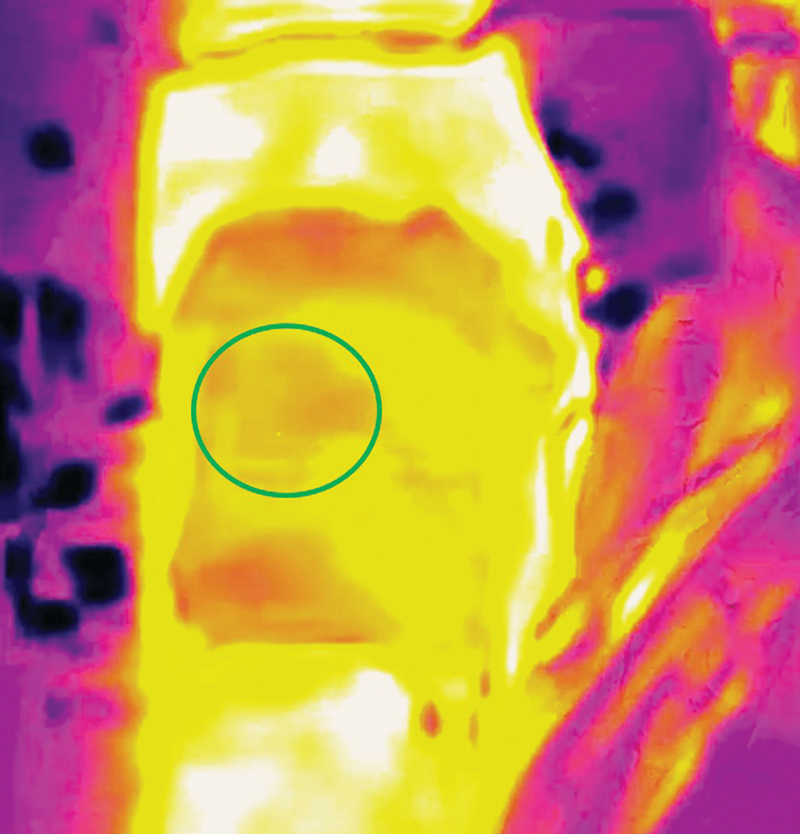
Smartphone-based thermal imaging. After cooling the anterolateral thigh, the perforasome shows as a hotspot (marked with the green circle).

Compact portable thermal cameras that can connect to smartphones can be used for pre-, per-, and postoperative imaging in flap surgery (local and free flaps). Based on the relation between tissue perfusion and skin temperature, these cameras allow the detection of “hotspots” on the skin that correspond with perforasomes. 2 However, it is not possible to visualize anatomical details of the course of the perforator. 31 The relatively low cost and non-invasive nature of the procedure are two important advantages 31 ( Fig. 14 ).

#### Lymphoscintigraphy

**Fig. 15:**
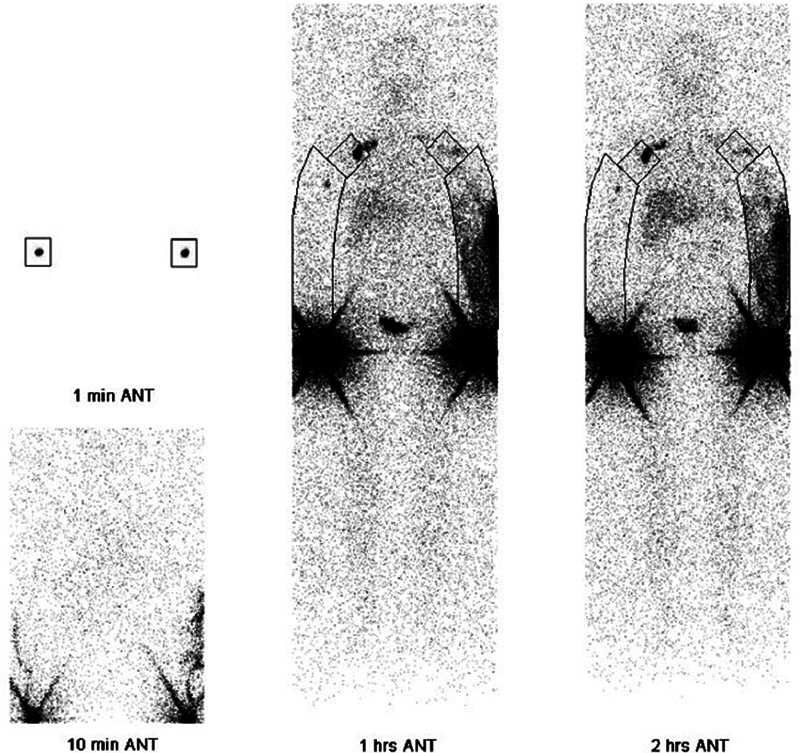
Lymphoscintigraphy upper extremities. Lymphatic fluid leakage into the subcutaneous tissue (dermal backflow) is seen in the left arm. Reduced uptake of 99m-technetium-labeled contrast agent is seen in the left axillary lymph nodes.

**Fig. 16:**
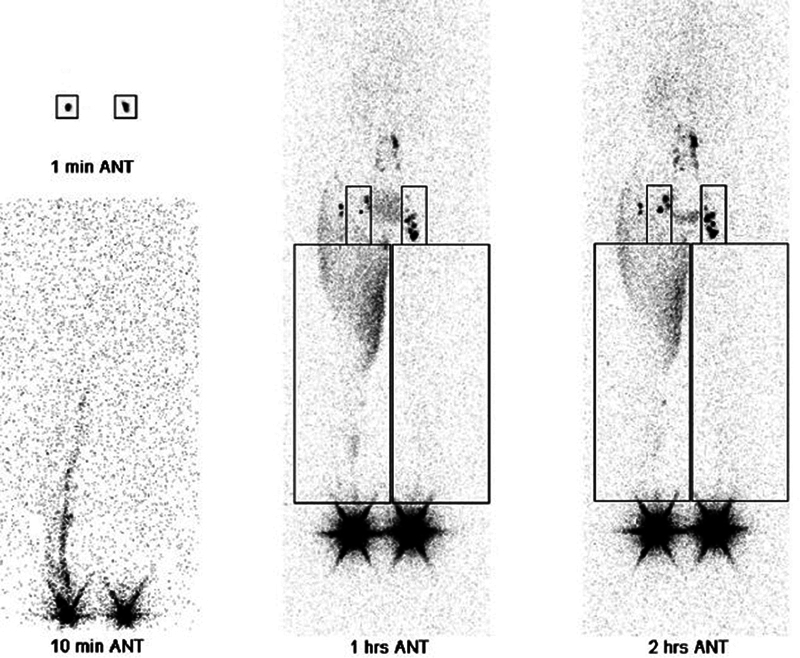
Lymphoscintigraphy lower extremities. Lymphatic fluid leakage into the subcutaneous tissue (dermal backflow) is seen in the right leg. Reduced uptake of 99m-technetium-labeled contrast agent is seen in the right inguinal lymph nodes.

Lymphoscintigraphy is the gold standard for assessing lymphatic function in lymphedema patients in order to decide if lymphatic surgery is indicated. It shows the lymphatic uptake of 99m-technetium-labeled contrast agent in the whole body with the help of a gamma camera. Subcutaneous or intradermal injections are performed. Intradermal injections show better visualization of the superficial lymphatics. 29 The transit time, the time needed for the 99m-technetium-labeled contrast agent to reach the proximal lymph nodes, can be measured. The information from the lymphoscintigraphy can be used in the staging of the severity of lymphedema. 29 Important parameters that play a role in diagnosis and staging are visualization of inguinal or axillary lymph nodes, lymphatic fluid leakage into the subcutaneous tissue (dermal backflow), and uptake in popliteal or antecubital lymph nodes 29 ( Figs. 15 and 16 ).

#### Contrast-Enhanced Magnetic Resonance Lymphography

**Fig. 17:**
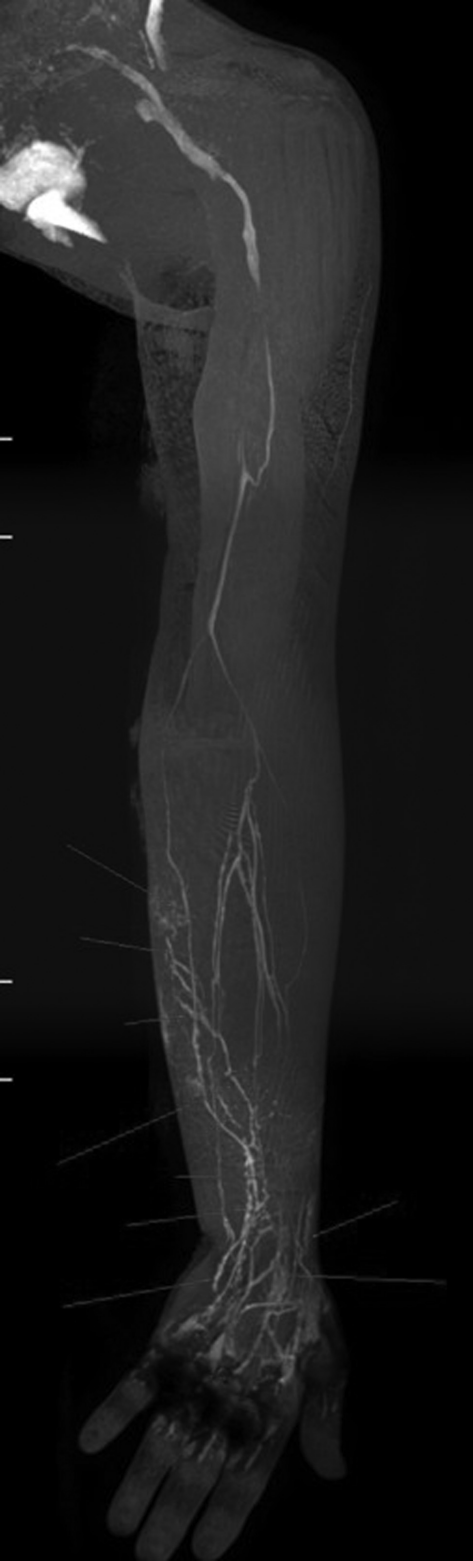
Contrast-enhanced magnetic resonance lymphography of the left upper extremity. The red arrows show the lymphatic vessels.

Contrast-enhanced MRL produces high-resolution imaging of superficial as well as deep lymphatic vessels. 2 An intracutaneous injection of gadolinium is used as a contrast agent. 2 Contrast-enhanced MRL is able to distinguish if the lymphatic vessels are dilated (in advanced stages of lymphedema) or not. However, veins are also simultaneously enhanced by the contrast agent and can only be differentiated from lymphatics based on their appearance or contrast agent uptake and clearance differences. 2 29 Affected lymphatic vessels have a beaded and tortuous look compared to the smooth veins. 29 Veins show an earlier enhancement and faster clearance of the contrast compared with lymphatic vessels. 29 Moreover, visualization of healthy lymphatic vessels seems to be very difficult because of the small diameter 29 ( Fig. 17 ).

#### Photoacoustic Imaging

Photoacoustic imaging is a more recent modality for evaluating the lymphatic vessels. It is not yet available for daily clinical practice. ICG is used, but the imaging is based on its optical absorption properties. Chromophores such as hemoglobin, ICG, and melanin absorb light of a certain wavelength. 29 This causes a thermoelastic expansion and generates acoustic waves that are detected by an ultrasound transducer. 28 Photoacoustic imaging shows functional lymphatics as well-defined linear patterns. 2 Winding patterns in photoacoustic imaging are comparable to splash patterns in near-infrared fluorescent lymphography. 2 Furthermore, cross-sectional images can be produced, illustrating the lymphatic capillaries located deep to diffuse more superficial patterns. 2

#### Laser Tomography

Microscope-integrated laser tomography is an experimental new technique that uses reflection of near-infrared light to provide images and generate high-resolution cross-sectional and optical coherence tomographic data. 32 By doing so, it enables real-time images of the lymphatic vessels in extremely high resolution. 32 This technique makes it possible to visualize functional lymphatic vessels with minimal to no sclerosis. 2 The diameter of the lumen and the thickness of the complete lymphatic vessel can be determined by using the cross-sectional images. 32 Furthermore, laser tomography can evaluate the patency of a lymphovenous anastomosis and even shows if inverted edges are present. 32 Inverted edges of vessels are an indicator to perform a redo of the anastomosis. However, the limited reaching distance of near-infrared light causes a disadvantage to this technique. The deepest layer from which the device can obtain images is 2.5 mm from the superficial surface after incision. 32 Therefore, preoperative examination is not possible.

### Comparison of Imaging Modalities

Tables 1 and 2 .

**Table 1:** Imaging modalities available for flap surgery planning

	CTA	MRA	HHD	CDU	UHFUS	Smartphone-based thermal imaging
Anatomy						
Information on course of the perforator	++	++		++ + +	++ + +	
Image depth	++ + +	++ + +	++	+++	+	+
Physiology						
Dynamic evaluation flow/pulsation			+	++ + +	++ + +	
Technical aspects						
Time-consuming for the surgeon			+	++ + +	++ + +	++
Technical skills to be learned by the surgeon			+	+++	+++	+
Cost	+++	++ + +	+	++	+++	+
Use of contrast	+	+				
Radiation exposure	+					

Abbreviations: CDU, color Doppler ultrasonography; CTA, computed tomography angiography; HHD, handheld Doppler; MRA, magnetic resonance angiography; UHFUS, ultrahigh-frequency ultrasound.

None to ++ + +, relative score, except for use of contrast and radiation exposure: none or +.

**Table 2:** Imaging modalities available for lymphatic surgery planning

	Lymphoscintigraphy	MRL	UHFUS	Near-infrared fluorescent lymphography (ICG)
Anatomy				
Accurate localization of the lymphatic vessel/lymph node		++	++ + +	+++
Anatomy of lymphatic vessel wall/lymph node			++	
Image depth	+++ (however, due to projection, depth information is lost)	++ + +	++ (up to 30 mm)	++ (up to 15 mm)
Ability to identify vein			+	
Physiology				
Dynamic evaluation flow	++ + +		++ a	+++
Peroperative confirmation of anastomosis patency				+
Technical aspects				
Time-consuming for the surgeon			++ + +	+
Technical skills to be learned by the surgeon			+++	+
Cost	+++	++ + +	++	++
Use of contrast	+	+		+
Radiation exposure	+			

Abbreviations: ICG, indocyanine green; MRL, contrast-enhanced magnetic resonance lymphography; UHFUS, ultrahigh-frequency ultrasound.

None to ++ + +, relative score, except for ability to identify vein/peroperative confirmation of patency anastomosis/use of contrast/radiation exposure, none or +.

a Using “superb microvascular imaging.” 33

### Imaging Work-Up: Case Report

**Fig. 18:**
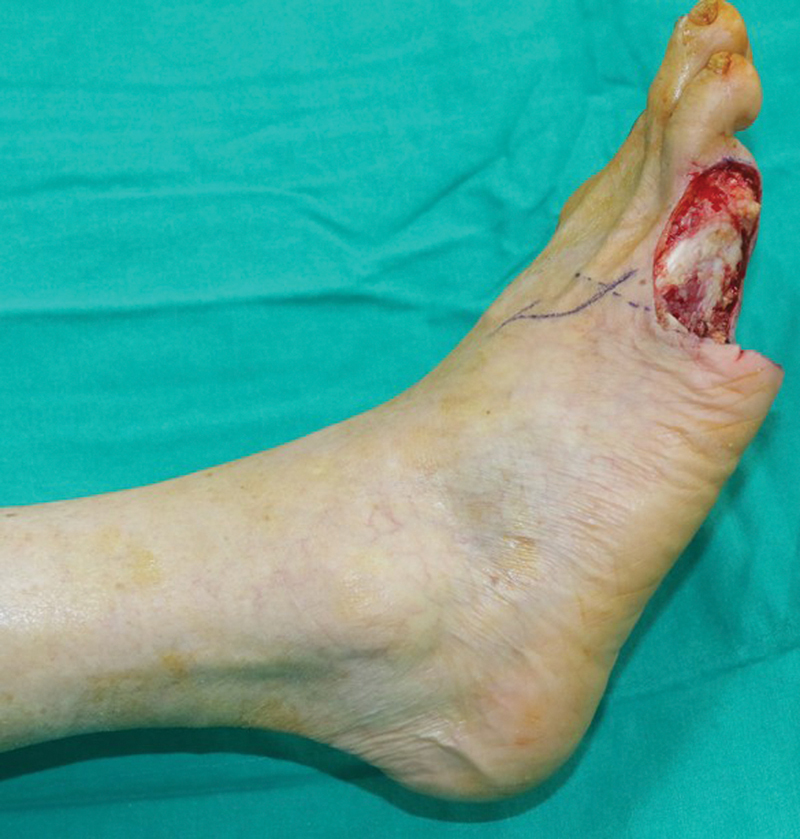
Intraoperative picture of the defect after resection.

A case of an oncologic resection with immediate reconstruction (ALT flap) is shown ( Figs. 18 19 20 21 22 23 24 25 ).

**Fig. 19:**
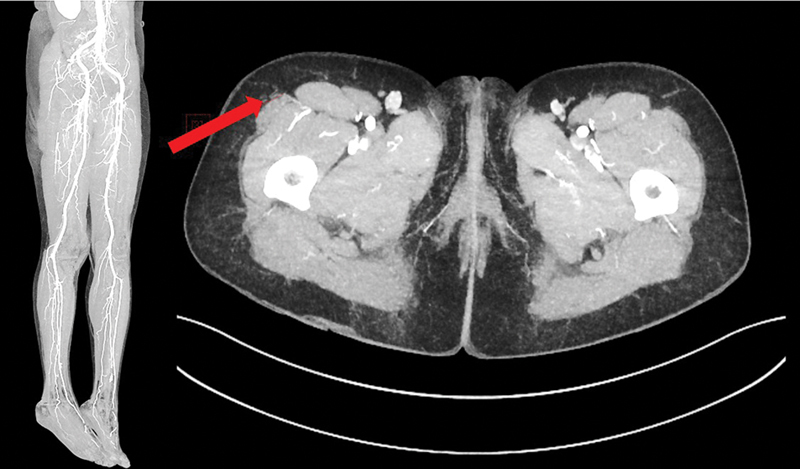
Preoperative computed tomography angiography shows patent lower extremity arteries (bilateral) and an adequate perforator of the descending branch of the lateral femoral circumflex artery of the right leg (red arrow).

**Fig. 20:**
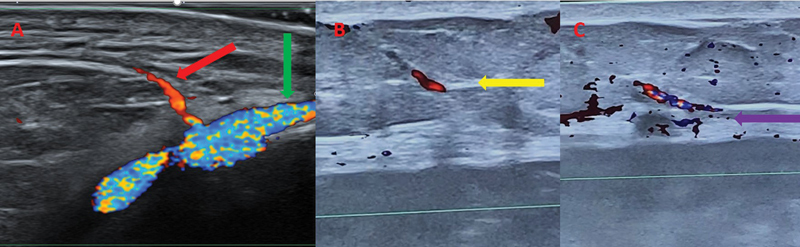
Preoperative color Doppler ultrasonography of the recipient site and anterolateral thigh flap. ( **A** ) Dorsal metatarsal artery (red arrow), branching from dorsalis pedis artery (green arrow). This course was marked on the foot of the patient (see Fig. 18 ). A dorsal metatarsal vein was traced as well (not shown in the figures). ( **B** ) Perforator piercing the superficial fascia (yellow arrow) of the anterolateral thigh. ( **C** ) Perforator piercing the deep fascia (purple arrow) of the anterolateral thigh. The mergence points of the perforator through the deep and superficial fascia are marked on the skin with a cross and a circle, respectively (see Fig. 21 ).

**Fig. 21:**
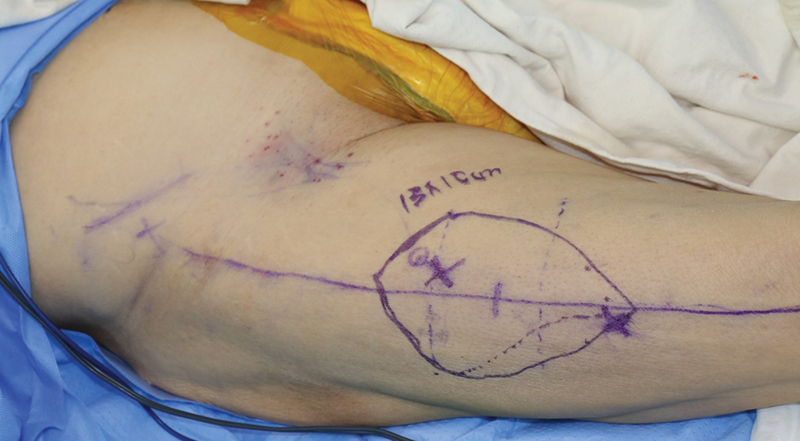
The mergence points of the perforator through the deep and superficial fascia are marked on the skin with a cross and a circle, respectively.

**Fig. 22:**
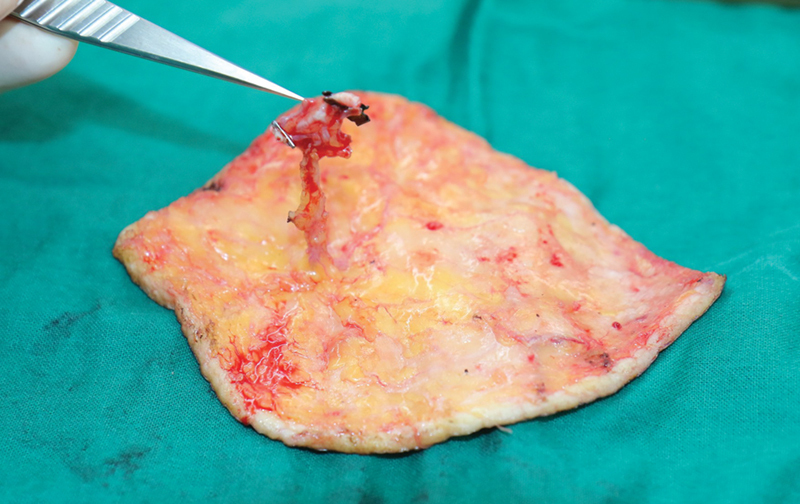
Ultrathin anterolateral thigh free flap was harvested.

**Fig. 23:**
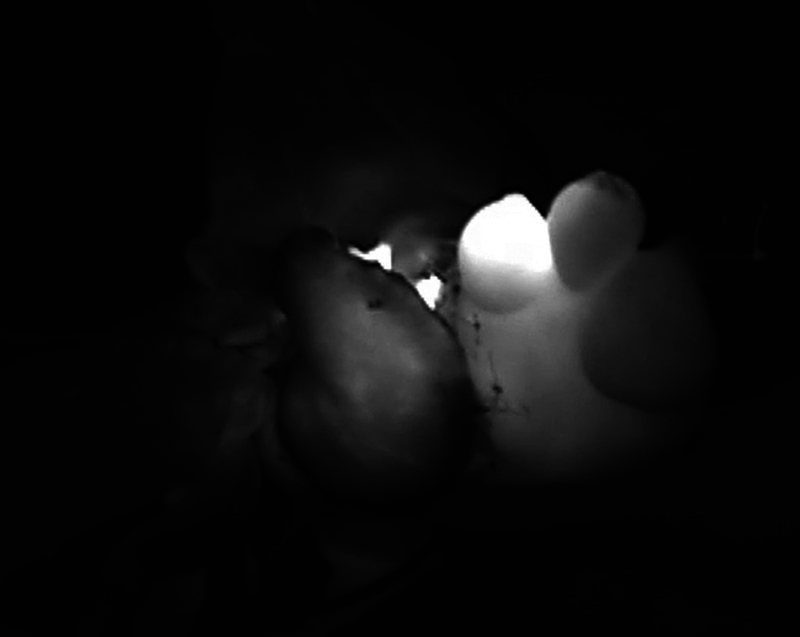
Microanastomosis was performed: Artery of the pedicle of the flap end-to-end to the dorsal metatarsal artery/1 concomitant vein of the pedicle of the flap end-to-end to a dorsal metatarsal vein. After intravenous injection of ICG, perfusion of the flap reconstruction is evaluated. ICG, indocyanine green.

**Fig. 24:**
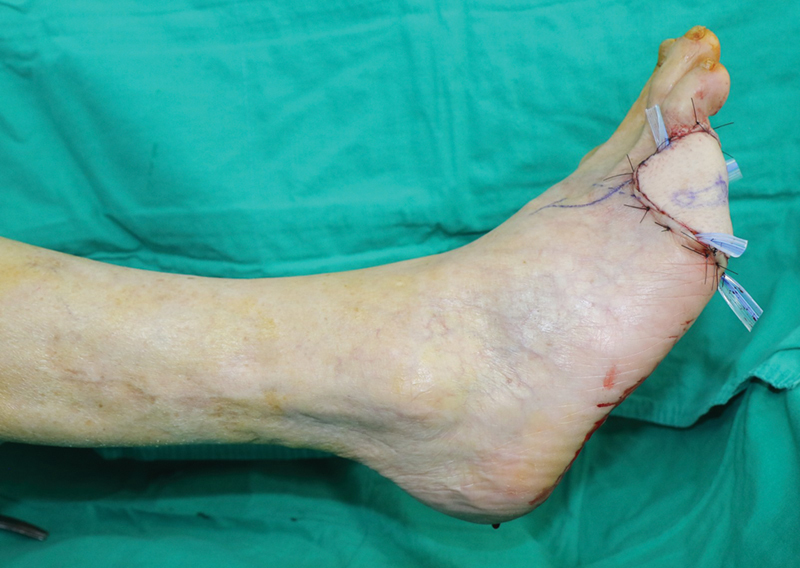
Intraoperative picture of the reconstruction.

**Fig. 25:**
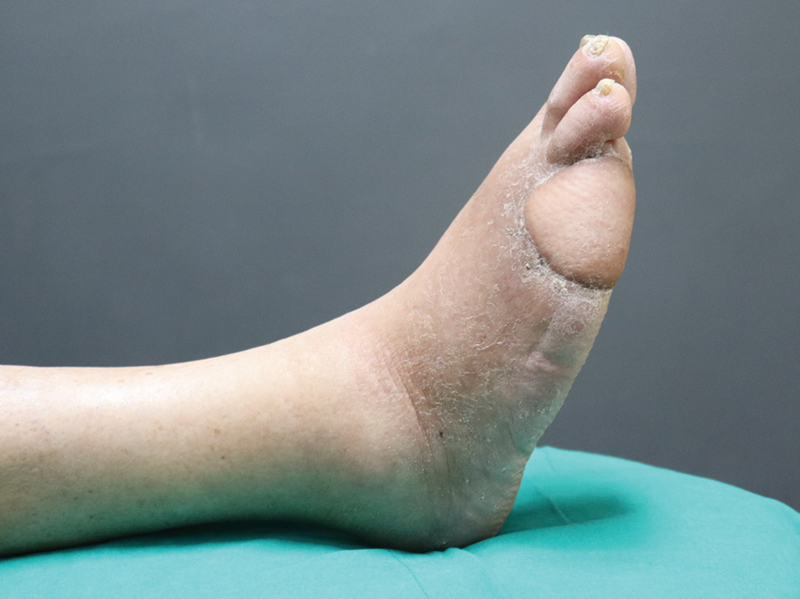
Picture of the healed reconstruction.

## Discussion

Advanced medical imaging is an indispensable tool in the planning and execution of supermicrosurgery, which critically depends on the precise identification of microvascular structures and functional lymphatic pathways. The research question “What are the state-of-the-art medical imaging modalities supporting supermicrosurgery?” was answered by performing a scoping review. Moreover, the article provides practical guidelines, compares the different imaging modalities for preoperative planning and intraoperative guidance, and illustrates the imaging work-up in daily practice with a case report.

### Vascular Imaging for Flap Surgery

In the realm of flap reconstruction, CTA remains the current gold standard for preoperative planning. CTA offers visualization down to vessel diameters of 0.3 mm and allows for the creation of 3D reconstructions. 4 MRA serves as a vital alternative, particularly for patients where radiation exposure is a concern, or due to its lower risk of acute allergic reactions associated with gadolinium compared to radioactive agents. 7 8 However, limitations of MRA for supermicrosurgery are the poorer resolution, only reliably visualizing vessels down to approximately 0.8 mm, and it is generally more time-consuming and expensive than CTA. 9 10 11

The rise of supermicrosurgery, emphasizing perforator-to-perforator anastomoses, necessitates detailed information about vessel course, size, and flow velocity. Here, high-frequency ultrasound and UHFUS have gained a major role. Combining UHFUS and probes with lower frequencies allows a detailed mapping of perforators up to the subdermal capillaries as well as major vessels and recipient sites. 14 It facilitates a 3D understanding of the vascular anatomy, essential for superthin skin flaps. The color mode displays the direction of the blood flow. Measuring physiological data, such as the flow velocity of recipient arteries, can be crucial, particularly in complex patients like those with ischemic diabetic foot. 14 Nevertheless, the utility of ultrasound is highly dependent on the operator; it is time-consuming, and it is often difficult to gain an overall view of an entire limb.

ICG angiography complements the other imaging techniques. Peroperatively, it can evaluate the patency of the anastomosis, as well as the perfusion of mastectomy skin or soft tissues after trauma.

Lastly, smartphone-based thermal imaging presents a low-cost, non-invasive method to locate perforasomes (“hotspots”) on the skin, although it lacks the anatomical precision needed to visualize the actual course of the perforator. 2 31

### Imaging for Lymphatic Surgery

Accurate imaging of the lymphatic and vascular system is paramount for surgical interventions like lymphovenous anastomosis and vascularized lymph node transfer, requiring identification of functional lymphatic vessels and lymph nodes as well as suitable recipient vessels.

Lymphoscintigraphy remains the gold standard for lymphedema diagnosis and severity staging. 29 Intradermal injections show better visualization of the superficial lymphatics compared to subcutaneous injections. 29 Lymphoscintigraphy provides information on the transit time, the time needed for the 99m-technetium-labeled contrast agent to reach the proximal lymph nodes; it visualizes inguinal or axillary lymph nodes, as well as lymphatic fluid leakage into the subcutaneous tissue (dermal backflow) and uptake in popliteal or antecubital lymph nodes. 29 However, the main limitation is the low resolution, which renders the precise localization of anastomosis sites unreliable for supermicrosurgery. 29

Near-infrared fluorescent lymphography, using intradermally injected ICG, gains prominence due to the good image quality and ease of use, providing real-time visualization of lymphatic vessels. 2 29 Lymphatic patterns can be recognized: Linear in functional lymphatics and splash, stardust, diffuse patterns in degenerated lymphatics. 2 29 Moreover, after performing a lymphovenous anastomosis, patency can be tested by ICG. The near-infrared fluorescent lymphography is also a tool to visualize the lymphatic vessels during the planning of an LyFT flap. 30 However, near-infrared fluorescent lymphography suffers from a limited depth penetration of 5 to 10 mm ^2^ .

The limitations of surface visualization are addressed by contrast-enhanced MRL. It produces high-resolution imaging of superficial as well as deep lymphatic vessels and makes an evaluation of the subcutaneous fat layer possible, offering an advantage in surgical planning. 2 However, veins are also simultaneously enhanced by the contrast agent and can only be differentiated from lymphatics based on their appearance or contrast agent uptake and clearance differences. 2 29 Affected lymphatic vessels have a beaded and tortuous look compared to the smooth veins. 29 Veins show an earlier enhancement and faster clearance of the contrast compared with lymphatic vessels. 29 Moreover, visualization of healthy lymphatic vessels seems to be very difficult because of their small diameter. 29

Lastly, high-frequency ultrasound and UHFUS can detect lymphatic vessels and surrounding venules based on shape, echogenic texture, color mode, collapsibility, and location. 19 20 The use of ultrasound addresses limitations of near-infrared fluorescent lymphography by enabling the detection of lymphatic vessels that are hidden by dermal backflow or lymphatic vessels situated in deeper tissue layers. 15 UHFUS is able to measure the size of lymphatic vessels. 15 19 Moreover, UHFUS makes it possible to evaluate the thickness of the lymphatic vessel wall in relation to the lumen in order to estimate the degree of lymphosclerosis. 14 The key disadvantages remain its high operator dependency and demanding learning curve.

### Novel and Experimental Techniques

Photoacoustic imaging and laser tomography represent promising, albeit mostly experimental, frontiers in lymphatic imaging. Photoacoustic imaging uses ICG's optical absorption properties. It shows functional lymphatics as well-defined linear patterns. 2 Winding patterns in photoacoustic imaging are comparable to splash patterns in near-infrared fluorescent lymphography. 2 Furthermore, cross-sectional images can be produced, illustrating the lymphatic capillaries located deep to diffuse more superficial patterns. 2 Microscope-integrated laser tomography provides real-time visualization of lymphatic vessels with extremely high resolution by using reflection of near-infrared light. 32 This technique makes it possible to visualize functional lymphatic vessels with minimal to no sclerosis. 2 The diameter of the lumen and the thickness of the complete lymphatic vessel can be determined by using the cross-sectional images. 32 Furthermore, laser tomography can evaluate the patency of a lymphovenous anastomosis and even shows if inverted edges are present. 32 However, the limited penetration depth of 2.5 mm causes restrictions and does not allow preoperative examination. 32

### Limitations

This study, conducted as a scoping review, highlights the current standards and emerging modalities. It also compares several techniques based on the available literature. However, a quantitative, head-to-head comparison of all modalities is difficult due to the heterogeneity of existing studies. Moreover, scoping reviews are subject to selection bias and publication bias. We focused on performing the analysis as objectively as possible, taking into account the results of the available studies. However, since studies with undesirable results might not be published, it is impossible to include unpublished insights in the scoping review.

## Conclusion

CTA and MRA are well-known standard imaging modalities in flap surgery. In lymphatic surgery, lymphoscintigraphy is the gold standard. However, in the era of supermicrosurgery, clinicians need more precise imaging modalities to know the exact microvascular and lymphatic anatomy of the patient. UHFUS has taken a major role in preoperative planning of flap surgery and lymphatic surgery. ICG angiography and near-infrared fluorescent lymphography have also become key elements in modern flap surgery and lymphatic surgery. Moreover, contrast-enhanced MRL produces high-resolution imaging of superficial as well as deep lymphatic vessels. Laser tomography and photoacoustic imaging are promising experimental imaging techniques in lymphatic surgery. This article describes and compares possible imaging modalities for preoperative planning and intraoperative guidance with the aims of enhancing surgical outcomes, reducing operative time, and preventing complications in supermicrosurgery. Moreover, a case report is described in order to illustrate the practical imaging work-up in daily practice.
